# Boosting Hydrogenation of CO_2_ Using Cationic Cu Atomically Dispersed on 2D γ‐Al_2_O_3_ Nanosheets

**DOI:** 10.1002/anie.202505444

**Published:** 2025-04-26

**Authors:** Ping Chen, Yifeng Zhu, Hailin Zhang, Micah P. Prange, Duo Song, Janos Szanyi, Yining Wang, Ying Chen, Xiang Wang, Oliver Y. Gutiérrez, Zihua Zhu, Zheming Wang, Carolyn I. Pearce, Ping Li, Kevin M. Rosso, Honghong Shi, Xin Zhang

**Affiliations:** ^1^ Physical & Computational Science Directorate Pacific Northwest National Laboratory Richland WA 99354 USA; ^2^ Institute of Process Engineering Chinese Academy of Sciences Beijing 100190 P.R. China; ^3^ Institute of Integrated Catalysis Pacific Northwest National Laboratory Richland WA 99354 USA; ^4^ Environmental Molecular Sciences Laboratory Pacific Northwest National Laboratory Richland WA 99354 USA

**Keywords:** 2D catalyst, CO_2_ hydrogenation, Cu/γ‐Al_2_O_3_, Methanol, Single‐atom catalysis

## Abstract

The continuous development of novel catalytic approaches is crucial for advancing efficient CO_2_ hydrogenation processes. Drawing inspiration from single‐atom catalysis and 2D materials, we designed a new 2D single‐atom catalyst with excellent thermal stability by thermally treating Cu‐adsorbed γ‐AlOOH nanosheets, which yielded a Cu/γ‐Al_2_O_3_ catalyst with high activity in the hydrogenation of CO_2_‐yielding methanol (CH_3_OH), dimethyl ether (DME), and CO as products. The active Cu sites are monodispersed and highly stable due to their cationic oxidation state and their substitution for pentacoordinated aluminum (Al^P^) sites on particle surfaces. This study demonstrates an efficient approach for achieving a high CO_2_ hydrogenation rate (30.45 mol mol^−1^ h^−1^) using a catalyst system that lacks metallic Cu centers, traditionally considered essential for H₂ dissociation, and employs what was previously thought to be an inert metal oxide (γ‐Al_2_O_3_) for CO and CH_3_OH production. Ongoing mechanistic studies aim to elucidate the synergy between cationic Cu single atoms and γ‐Al_2_O_3_, a Lewis acid support, in facilitating hydrogen (H_2_) activation and methanol formation.

## Graphical abstract

A novel 2D single‐atom catalyst featuring cationic Cu atomically dispersed on γ‐Al₂O₃ nanosheets has been developed, demonstrating exceptional thermal stability and catalytic activity for CO₂ hydrogenation. Compared to traditional Cu/ZnO/Al₂O₃ systems, this catalyst achieves higher methanol (CH_3_OH) and dimethyl ether (DME) production rates, driven by the unique substitution of Cu for pentacoordinated Al sites. This innovative design enhances H₂ activation and methanol synthesis, offering new insights into single‐atom catalysis for sustainable CO₂ utilization.

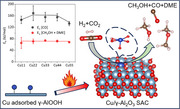

The hydrogenation of CO_2_ to methanol (CO_2_ + 3H_2_ → CH_3_OH + H_2_O) is recognized as one of the most effective and economical methods for fixing and utilizing large amounts of emitted CO_2_. Optimizing the efficiency of this reaction is an attractive route to sustainability because it converts CO_2_, a cheap, nontoxic, and abundant C1 feedstock, into high‐value‐added chemicals and energy fuels as alternatives to petroleum.^[^ ^1^, ^2^, ^3^ ^]^ At present, commercially recognized benchmark catalysts with the highest CH_3_OH selectivity are Cu/ZnO_2_/Al_2_O_3_ or Cu/ZrO_2_/Al_2_O_3_.^[^ ^4^, ^5^, ^6^, ^7^, ^8^, ^9^, ^10^ ^]^ In these systems, metallic Cu nanoparticles are typically considered the active sites for H_2_ activation, while metal oxides such as ZnO_2_ and ZrO_2_ facilitate strong CO_2_ adsorption. Al_2_O_3_ is generally viewed as an inert support, playing a role in enhancing the physical stability and dispersion of ZnO or ZrO_2_ species. The specific role of Cu in these catalysts, whether in its metallic form (Cu^0^) or in cationic states (Cu^+^, Cu^2+^), as the active site for methanol synthesis remains under debate. Previous studies suggest catalytic activity is proportional to the surface area of exposed Cu^0^ produced by reduction prior to the reaction because H_2_ dissociation cannot be performed in an efficient manner on Cu cations or on the oxide.^[^ ^11^, ^12^, ^13^ ^]^ In addition, deactivation studies of the Cu/ZnO/Al_2_O_3_ catalyst >720 h on‐stream have shown that deactivation is primarily due to the partial oxidation of Cu^0^ to Cu^2+^ and the slight agglomeration of ZnO species during long‐term operation,^[^ ^14^ ^]^ suggesting that Cu^2+^ contributes reversely to hydrogenation of CO_2_ compared with the Cu^0^.

Alternatively, other studies highlight the importance of the metal‐oxide interface,^[^ ^15^, ^16^, ^17^, ^18^ ^]^ suggesting that positively charged Cu species (Cu–O–Zn) at the interface between Cu nanoparticles and the oxide are the true active sites for CO_2_ activation/hydrogenation, which favors the formation of methoxy species—the rate‐determining step for CO_2_‐to‐CH_3_OH.^[^ ^19^, ^20^ ^]^ In contrast to commercial, complex Cu metal particle systems that feature a mixture of metallic Cu, peripheral copper cations, and Cu–Zn alloys,^[^ ^21^ ^]^ the single‐atom Cu catalysts supported on a single oxide surface provide a simpler, more uniform system of separated Cu cations.^[^ ^1^ ^]^ They also offer an excellent model system for studying a homogeneous metal–metal oxide interface and are particularly suited to providing fundamental insights into the reaction of CO_2_ hydrogenation.^[^ ^1^ ^]^

Considerable evidence suggests that the outstanding performance of single‐atom catalysts (SACs) stems from a significant alteration in the electronic environment surrounding the central metal.^[^ ^22^, ^23^, ^24^ ^]^ For instance, the Cu SACs are more efficient than copper clusters or nanoparticles in dissociating hydrogen and activating CO_2_ to form HCOO*, a key intermediate for CH_3_OH synthesis, leading to high selectivity for CH_3_OH.^[^ ^1^ ^]^ In fact, the electronic environment around the single atoms is not only determined by the choice of metal‐support pair but also by the morphology and homogeneity of the support.^[^ ^25^, ^26^ ^]^ Changing the anchoring site of single atoms from terraces, steps, vacancies, facets, and kinks should result in different surface energy, therefore affecting the catalyst's reactivity.^[^ ^27^, ^28^ ^]^ Doping metals onto the 3D‐shaped materials usually results in a mixture of all these sites and the homogeneity of the surface can hardly be realized. For this reason, the concept of 2D‐material‐supported SACs emerged to maintain the uniformity of the surface to the largest extent. These 2D material supports also offer large surface area, ease of surface defect formation, and the possibility of uniformly controlled surface functionalization for tuning the spatial confinement of SACs and the design of active sites on the surface. Although systematic research on 2D materials‐derived catalysts is still lacking, there have been reports about the superior catalytic properties of metal doping of these 2D materials, especially in electrochemical or energy storage applications.^[^ ^29^ ^]^ So far, most single atom‐doped low dimensional materials reported in literature use carbon nanomaterials such as 0D fullerene, 1D carbon nanotubes, and graphene substrates.^[^ ^30^ ^]^ However, little work has been done on developing 2D‐shaped metal oxide‐supported SACs. To establish this route, here we report a facile two‐step synthesis method to obtain atomically dispersed Cu on γ‐Al_2_O_3_ nanosheets and evaluate its performance for CO_2_ hydrogenation. γ‐Al_2_O_3_ was selected as the support due to its high surface area (typically between 50 and 350 m^2^ g^−1[^ ^31^ ^]^), excellent mechanical and thermal stability (with no significant phase transformation below 700 °C^[^ ^32^ ^]^), and its widespread use in industrial catalysts as a structural promoter to enhance the dispersion of active metal or metal oxide species.^[^ ^33^, ^34^, ^35^, ^36^, ^37^ ^]^ The use of Cu‐adsorbed γ‐AlOOH nanosheets enables controlled deposition of Cu species, promoting the formation of highly dispersed and catalytically active Cu sites on the final γ‐Al_2_O_3_ surface. This structural design aims to optimize catalytic performance by improving both the dispersion and accessibility of the active Cu species.

First, boehmite (γ‐AlOOH) with a high specific surface area (375 m^2^ g^−1^, Table S1, Supporting Information) and single‐layered structure was prepared, which was conducive to improving the adsorption capacity of copper ions. Varying amounts of Cu^2+^ cations were adsorbed onto boehmite particle surfaces via aqueous solution exposure (initial concentration of Cu precursor solution: 0.5–10 wt%). Next, the Cu^2+^ adsorbed γ‐AlOOH was converted to Cu/γ‐Al_2_O_3_ by calcination in air at 600 °C. This led to various Cu/γ‐Al_2_O_3_ catalysts with Cu loading ranging from 0.24 to 0.52 wt% based upon inductively coupled plasma (ICP) analyses. The prepared catalysts were pretreated at 250 °C in 20 vol% H_2_/N_2_ for 30 min prior to catalytic testing. This pretreatment primarily increases the surface concentration of hydrogen species, thereby preparing the surface for the subsequent catalytic reaction. To avoid misinterpretation of the morphology or nature of the active Cu species under reaction conditions—such as potential agglomeration of surface Cu species in H₂ at elevated temperatures—the catalysts (Cu/γ‐Al_2_O_3_) were subjected to the same pretreatment protocol prior to characterization. After pretreatment, the catalysts were evaluated in CO_2_ hydrogenation in a fixed‐bed flow reactor at commercial working conditions (250–350 °C and 32 bar), producing CO, CH_3_OH, and DME as the main products. All the data were collected at CO_2_ conversion conditions far away from equilibrium (<5%), with the catalytic reaction being kinetically controlled. Since DME is likely formed from the secondary reaction of CH_3_OH dehydration on the Lewis acid sites of the Al_2_O_3_ support,^[^ ^38^ ^]^ we presented the CH_3_OH formation rate on the combined formation rate of CH_3_OH and DME, expressed in the form of turnover frequency (TOF) with unit of h^−1^ (Table S2 and Figure S1, Supporting Information). The catalyst that demonstrated the highest CH_3_OH + DME rate was found to have Cu loading as 0.49 wt%; henceforth this sample will be denoted as the Cu/γ‐Al_2_O_3_ with its precursor before calcination denoted as Cu/γ‐AlOOH and is the subject of detailed characterization in the rest of this paper. The performance of the Cu/γ‐Al_2_O_3_ in this work is compared with the Cu/ZnO/Al_2_O_3_ commercial catalyst tested on the same setup (as benchmark), and other reported Cu nanoparticle and SACs from the literature as listed in Table 1. The CH_3_OH + DME formation rate (TOF) of the Cu/γ‐Al_2_O_3_ catalyst developed in this work was found to be approximately one order of magnitude higher than that of the commercial Cu/ZnO/Al_2_O_3_ catalyst and three times higher than that of the state‐of‐the‐art Cu_1_/ZrO_2_ SAC (CAZ‐1). However, the CO selectivity remains significant (∼82%), indicating that the reverse water‐gas shift reaction (RWGSR) remains the predominant reaction pathway on the Cu/γ‐Al₂O₃ catalyst. And a detailed summary of the CO_2_ hydrogenation performance of various catalysts has been reported in many references,^[^ ^39^, ^40^, ^41^, ^42^, ^43^ ^]^ which emphasizes the advantages of our catalyst in terms of performance, synthesis method, and cost.

**Table 1:** Catalytic performance of metal oxide‐supported Cu nanoparticles and single atoms on the hydrogenation of CO_2_.

Catalysts	Conditions (*T*/°C, *P*/bar)	H_2_/CO_2_	CO rate (h^−1^)	CH_3_OH + DME rate (h^−1^)
Cu/ZnO/Al_2_O_3_ ^a)^ ^[^ ^44^ ^]^	240, 96.5	3	n. a.	0.47
Cu/ZnO/Al_2_O_3_ ^a)^ ^[^ ^45^ ^]^	240, 20	3	n. a.	0.65
Cu/ZnO/Al_2_O_3_ ^b)^ (this work^c)^)	250, 32	3	6.90	0.54
CAZ‐1(Cu_1_/ZrO_2_)^b)^ ^[^ ^1^ ^]^	180, 30	3	∼0	1.4
Cu_1_/ZnO^b)^ ^[^ ^46^ ^]^	170, 30	3	0.014	0.16
Cu/γ‐Al_2_O_3_ ^b)^ (this study^c)^)	170, 32	3	0	0.25
	250, 32	3	25.10	5.35

^a)^ Refers to Cu nanoparticle.

^b)^ Refers to Cu single atoms.

^c)^ Reaction conditions: 250 °C, 32 bar, CO_2_/H_2_/N_2_ = 7/21/1 mL min^−1^; the commercial Cu/ZnO/Al_2_O_3_ used as reference in this work are Johnson Matthey (JM) KATALCO 51 series.

n. a.: Refers to not available.

Powder X‐ray diffraction (XRD) patterns (Figure 1a) indicated that the synthesized γ‐AlOOH precursor substrate exhibits peaks consistent with the boehmite phase, aligning well with the γ‐AlOOH reference (JCPDS 21‐1307). The broadening of the (002) peak suggests a low degree of crystallinity. After calcination, the pattern aligns with the γ‐Al_2_O_3_ phase (JCPDS 00‐010‐0425), showing prominent peaks at 37.6°, 45.9°, and 67.0° corresponding to the (311), (400), and (440) planes. The crystal structure of γ‐AlOOH remains unchanged upon copper adsorption (Cu/γ‐AlOOH), and the thermal transformation products (Cu/γ‐Al_2_O_3_) mirror those of pure γ‐Al_2_O_3_, with no distinct Cu‐related peaks observed. The BET results (Table S1, Supporting Information) indicate that the specific surface area of γ‐AlOOH showed little change before and after Cu adsorption (from 349 to 332 m^2^ g^−1^). However, compared to the significant reduction in specific surface area after thermal calcination of the original γ‐AlOOH (from 349 to 185 m^2^ g^−1^), the specific surface area of Cu/γ‐AlOOH did not decrease significantly after heat treatment (from 332 to 282 m^2^ g^−1^). This suggests that the surface‐adsorbed Cu may undergo structural changes during the heat treatment process. This change helps mitigate the reduction in specific surface area, thereby facilitating the preparation of catalysts with high specific surface areas.

**Figure 1:**
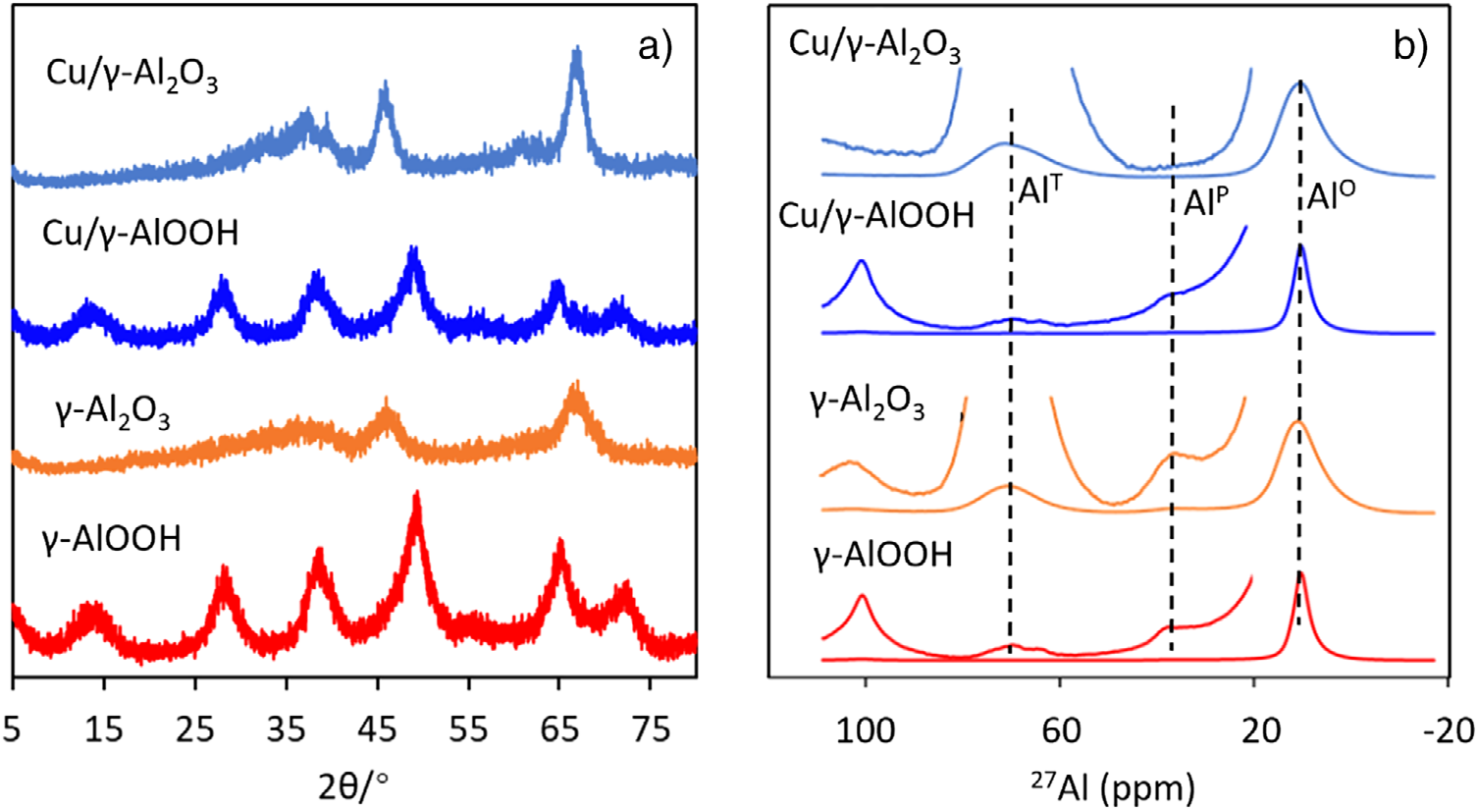
XRD patterns a), and ^27^Al MAS NMR spectra b), of 2D γ‐AlOOH, γ‐Al_2_O_3_, Cu/γ‐AlOOH, and Cu/γ‐Al_2_O_3._

In ^27^Al magic‐angle spinning nuclear magnetic resonance (MAS NMR) spectra of γ‐AlOOH and γ‐Al_2_O_3_ samples (Figure 1b), a major peak was located at about 10.8 ppm, and two low‐intensity peaks located at 36.6 and 70.1 ppm were observed. These peaks are assigned to octahedral aluminum coordination (Al^O^) (10.8 ppm), pentahedral aluminum coordination (Al^P^) (36.6 ppm), and tetrahedral aluminum coordination (Al^T^) (70.1 ppm),^[^ ^47^, ^48^ ^]^ respectively. Both Al^P^ and Al^T^ in γ‐AlOOH, as well as Al^P^ in γ‐Al_2_O_3_ represent structural incompleteness in the form of defects.^[^ ^48^, ^49^ ^]^ The ratio (mol%) of Al^O^, Al^P^, and Al^T^ are quantified and listed in the Table S3, Supporting Information. Compared to the γ‐AlOOH, the proportion of Al^O^ site decreased from 99.1% to 76.4%, while the Al^T^ sites increased from 0.4% to 22.9% in the γ‐Al_2_O_3_, indicating a lattice structure transformation from Al^O^ to Al^T^.^[^ ^50^ ^]^ It can be concluded that Cu adsorption does not affect the Al sites on the AlOOH significantly. However, as observed by comparing to the γ‐Al_2_O_3_ sample, Cu/γ‐Al_2_O_3_ catalyst showed a significantly decreased ratio of Al^O^ (from 76.4% to 70.5%) and Al^P^ (from 0.7% to 0.1%). Correspondingly, the ratio of Al^T^ sites increased from 22.9% to 29.4% in the Cu‐doped γ‐Al_2_O_3_ sample. These changes suggest that thermal treatment at 600 °C plays a crucial role in anchoring Cu atoms and modifying the surface Al species, where Cu atoms likely replaced the Al^O^ and/or Al^P^ sites.

X‐ray photoelectron spectroscopy (XPS) is widely used for studies of surface chemistry, offering a range of useful information on depths, reasonable quantification, and chemically specific details for each detected element through chemical shifts. The O 1s peaks (Figure 2a and Table S4, Supporting Information) are composed of subpeaks with peak energy ∼530.7, ∼532.1, and 533.3 eV, which can be designated as surface O^2−^, ─OH group, and ─H_2_O group,^[^ ^51^, ^52^ ^]^ respectively. The most noticeable change is the sharp increase in the amount of adsorbed water on samples containing Cu, indicating that copper enhances the surface activity. The binding energy of Al 2p (Figure 2b) shifted slightly from 74.3 to 74.7 eV^[^ ^53^ ^]^ and the peak shape was also affected due to the addition of Cu. This indicates the coordination environment for Al atoms has been changed after the introduction of surface Cu species, although the dominating chemical state of Al atoms in the Cu‐doped γ‐AlOOH and γ‐Al_2_O_3_ is still Al^3+^. The Cu 2p spectrum (Figure 2c) of Cu/γ‐AlOOH could be resolved to two peaks at 933.4 and 953.4 eV, corresponding to Cu^2+^ indicating that copper has been adsorbed onto the surface.^[^ ^54^, ^55^ ^]^ And Cu 2p spectrum of Cu/γ‐Al_2_O_3_ was also deconvoluted into two peaks at different binding energies of 933.4 and 953.4 eV, indicating that Cu^2+^ species were not reduced, with no presence of Cu^0^ or Cu^+^ species on the catalyst.^[^ ^56^, ^57^ ^]^ In addition, the nature of Cu^2+^ cannot be classified as Cu(OH)_2_, as evidenced by the absence of the two satellite peaks at 942.19 and 962.45 eV.^[^ ^58^ ^]^

**Figure 2:**
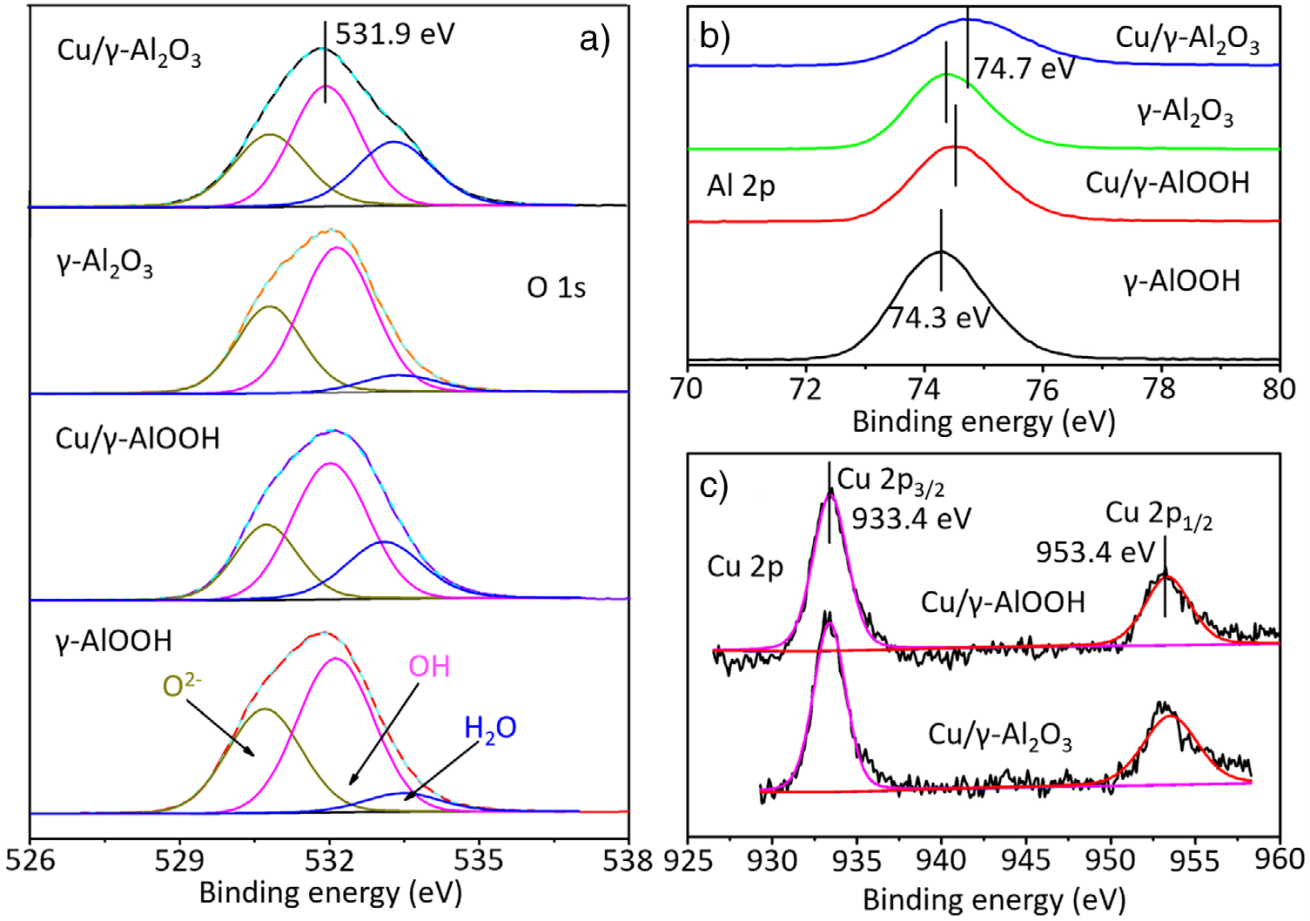
XPS spectra a), O 1s; b), Al 2p; c), Cu 2p of 2D γ‐AlOOH, γ‐Al_2_O_3_, Cu/γ‐AlOOH, and Cu/γ‐Al_2_O_3._

Transmission electron microscopy (TEM) images (Figure S2a,b, Supporting Information) reveal that both γ‐AlOOH and γ‐Al_2_O_3_ exhibit thin sheet‐like morphology with particle sizes ranging from 1 to 3 nm, and the addition of copper does not significantly alter these characteristics (Figures 3a and S2c, Supporting Information). Scanning transmission electron microscopy (STEM) of the Cu/γ‐Al_2_O_3_ in Figure 3b,c and the energy dispersive X‐ray (EDS) elemental mapping results in Figure 3d–g demonstrated that a uniform distribution of Cu atoms across particle surfaces is maintained after thermal transformation. Consistent with this picture, in our time‐of‐flight secondary ion mass spectrometry (ToF‐SIMS) observations (Figure S3, Supporting Information), neither Cu/γ‐AlOOH nor Cu/γ‐Al_2_O_3_ show a significant concentration of CuOCu^+^ species; instead, Cu exists primarily as CuOAl^+^ and Cu^+^, indicating that copper is predominantly in the form of single atoms. However, in Cu/γ‐Al_2_O_3_, the CuOAl^+^/Cu^+^ ratio changes significantly compared to Cu/AlOOH, increasing from the original 36.7% to 149.6%. This suggests that after the thermal phase transition from Cu/γ‐AlOOH to Cu/γ‐Al_2_O_3_, the coordination environment of Cu undergoes a fundamental transformation, shifting from an adsorbed state to a structurally steady state, which results in a much higher CuOAl^+^/Cu^+^ ratio. To further confirm the nature of surface Cu species and examine the atomic dispersion of Cu atoms in the studied catalyst, IR spectroscopy of CO adsorption on Cu/γ‐Al_2_O_3_ was collected with results shown in Figures 3h and S4, Supporting Information. From the collected spectra, we didn't observe peaks corresponding to *CO adsorbed on metallic Cu (Cu^0^), which typically shows peaks in the range of 2040–2120 cm^−1^, originating from the π‐backdonation from Cu to the CO antibonding orbital. In the CO adsorption region, two bands at ∼2160 and ∼2190 cm^−1^ originated from the CO adsorption on the γ‐Al_2_O_3_ support: the band at ∼2190 cm^−1^ is assigned to CO adsorbed on surface Lewis acid sites (Al^3+^), while the ∼2160 cm^−1^ band corresponds to CO hydrogen bonded to Al–OH groups^[^ ^59^, ^60^, ^61^ ^]^ (Figure S4, Supporting Information warming up from liquid N_2_ temperature to room temperature after CO adsorption). Both Cu^+^ and Cu^0^ sites form carbonyls that, when copper is highly dispersed, show CO bands at similar frequency (∼2136 cm^−1^) as Cu^+^–CO or Cu^0^–CO carbonyls. However, the two species can be distinguished by their stability: Cu^0^–CO species are easily decomposed during evacuation, whereas CO remains strongly adsorbed on Cu^+^ sites.^[^ ^62^, ^63^, ^64^ ^]^ In this work, only a significant peak at 2136 cm^−1^ was observed after evacuation (Figure 3h), corresponding to strong chemisorption of CO* adsorbed on the top of Cu cations (Cu^+^). Notably, the absence of CO adsorption bands associated with Cu^0^ nanoparticles (2040–2120 cm^−1^) confirms the exclusive presence of cationic Cu species in our catalyst system. This observation provides direct spectroscopic evidence for the unique metal‐support interactions in our Cu/γ‐Al_2_O_3_ SAC, where the γ‐Al_2_O_3_ nanosheets play a crucial role in stabilizing the highly dispersed Cu atoms.

**Figure 3:**
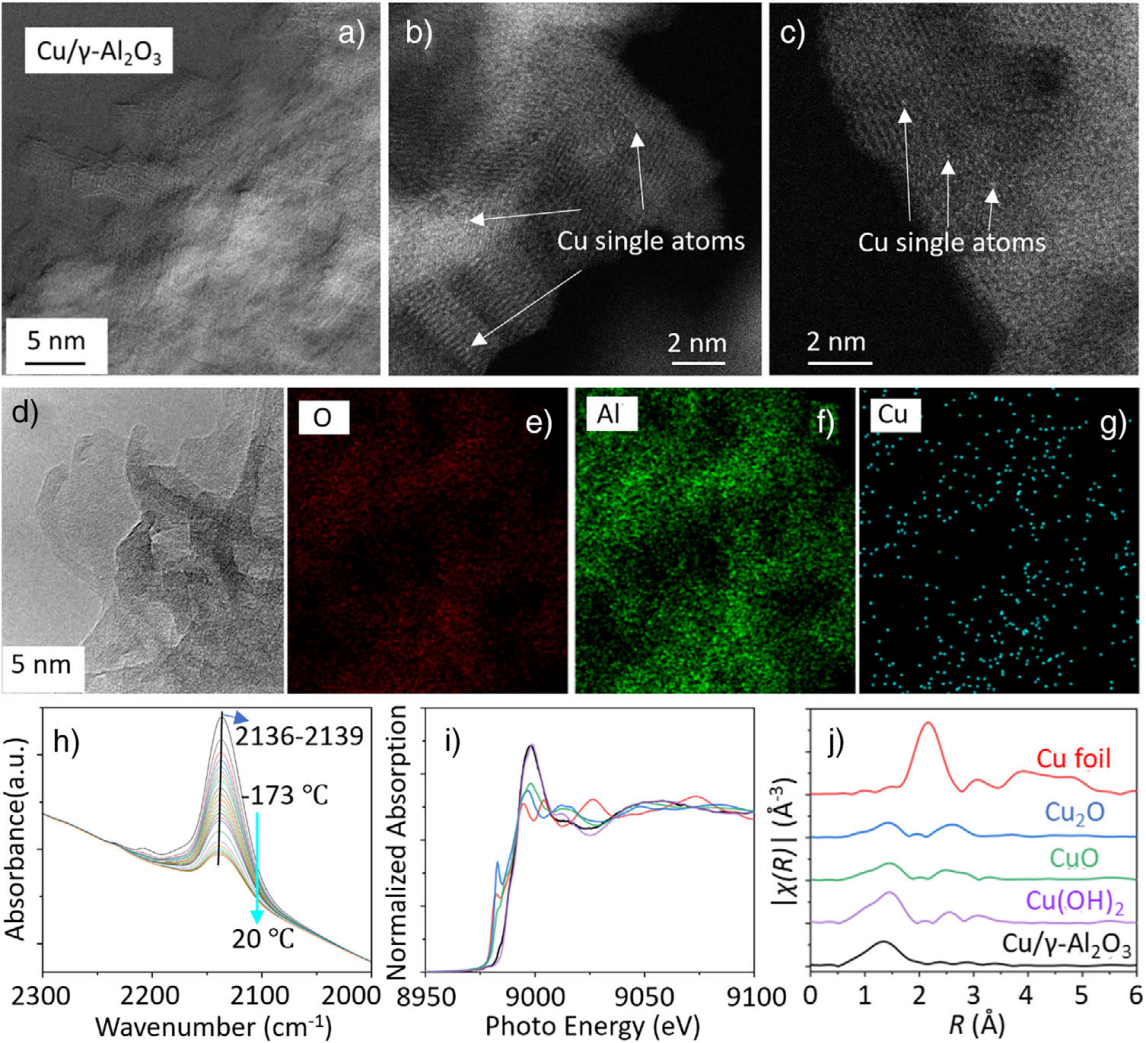
a) TEM image of Cu/γ‐Al_2_O_3_. b–d) HAADF‐STEM image of Cu/γ‐Al_2_O_3_. e–g) STEM‐EDS elemental mapping images Cu/γ‐Al_2_O_3_. h) FTIR spectra of evacuation on Cu/γ‐Al_2_O_3_ at room temperature after CO adsorption at −173 °C (Figure S4a) and heating to 9 °C (Figure S4b). i–j) Cu K‐edge XANES spectra and Cu K‐edge EXAFS spectra of Cu/γ‐Al_2_O_3_, and Cu standards (Cu foil, Cu_2_O, CuO, and Cu(OH)_2_); fitting and wavelet transform analysis results are shown in the Supporting Information.

Figure 3i shows normalized Cu K‐edge XANES spectra for the H_2_ pretreated Cu/γ‐Al_2_O_3_ sample and the different copper standard compounds. The pre‐edge feature of Cu originates from the transition to spatially localized 3d states, and the shape of the pre‐edge depends on the number of electrons in the d‐shell and its intensity is proportional to the amount of 3d–4p hybridization.^[^ ^65^ ^]^ The sharp shoulder on the rising edge for Cu is known as indicative of a linear or square planar geometry with a lower energy of empty 4p orbitals perpendicular to the chemical bonds. This shoulder appears in the spectra of Cu_2_O (linear, 2‐coordinated) or CuO (planar, 4‐coordinated) standards as expected (Figure 3i), but not in the Cu/γ‐Al_2_O_3_ sample. In addition, the absorption edge position, and the intensity of Cu/γ‐Al_2_O_3_ sample's white line are at nearly the same level of the Cu(OH)_2_. Therefore, we can infer that the Cu has coordination environment similar to Cu(OH)_2_ that has the oxidation state around 2+ and a pentahedral coordination with oxygen.

The Fourier transforms (FTs) of the EXAFS (extended X‐ray absorption fine structure) for the analyzed Cu/γ‐Al_2_O_3_ catalyst and reference samples are presented in Figure 3j, uncorrected for the phase change. It is apparent that the Cu–O coordination is the dominant coordination environment in the first shell. Uniting these characterization results, we obtained from the XPS, Al‐NMR, TOF‐SIMS, and XAS analysis of the H_2_‐pretreated Cu/γ‐Al_2_O_3_, we arrive at a geometry model that the surface copper species replaced Al atoms at the pentahedral sites on the surface (Al^P^) for EXAFS fitting and this was supported by our DFT optimization results (Figure 4a,b and Table S6, Supporting Information). Further details regarding this fitting are available in Figures S5, S6 and Table S5, Supporting Information. The attempts to replace Al^T^ or Al^O^ with Cu cations did not yield successful EXAFS fitting results. Consequently, the data was fitted up to 3.4 Å at a satisfactory level and the results proved our hypothesis on the surface structure. The fitting results revealed strong evidence that Cu atoms replaced the Al^P^ sites that demonstrated a nearly 5‐coordinated structure with Cu─O bond lengths of 2.0 Å (coordination number (CN) of Cu–O: 3.3 ± 0.8 and Cu─O bond lengths of 2.3 Å (CN of Cu–O: 0.8 ± 0.2), respectively. There is one Cu─O bond length longer than the other four Cu─O bonds, indicating the partial hydroxylation of the Cu site forming Cu–OH after hydrogen pretreatment. Additionally, the Cu─O bond distance is slightly longer than the Al^T^─O bond (∼1.8 Å), and we observed the presence of Cu–O–Al at 2.8 Å with a CN of ∼4. This value was derived from either the Cu–O–Al^O^ distance or the Cu–O–Al^P^ distance. The Cu–O–O triangular path and Cu–Al^T^ path were also found in the fitting of the higher shells.

**Figure 4:**
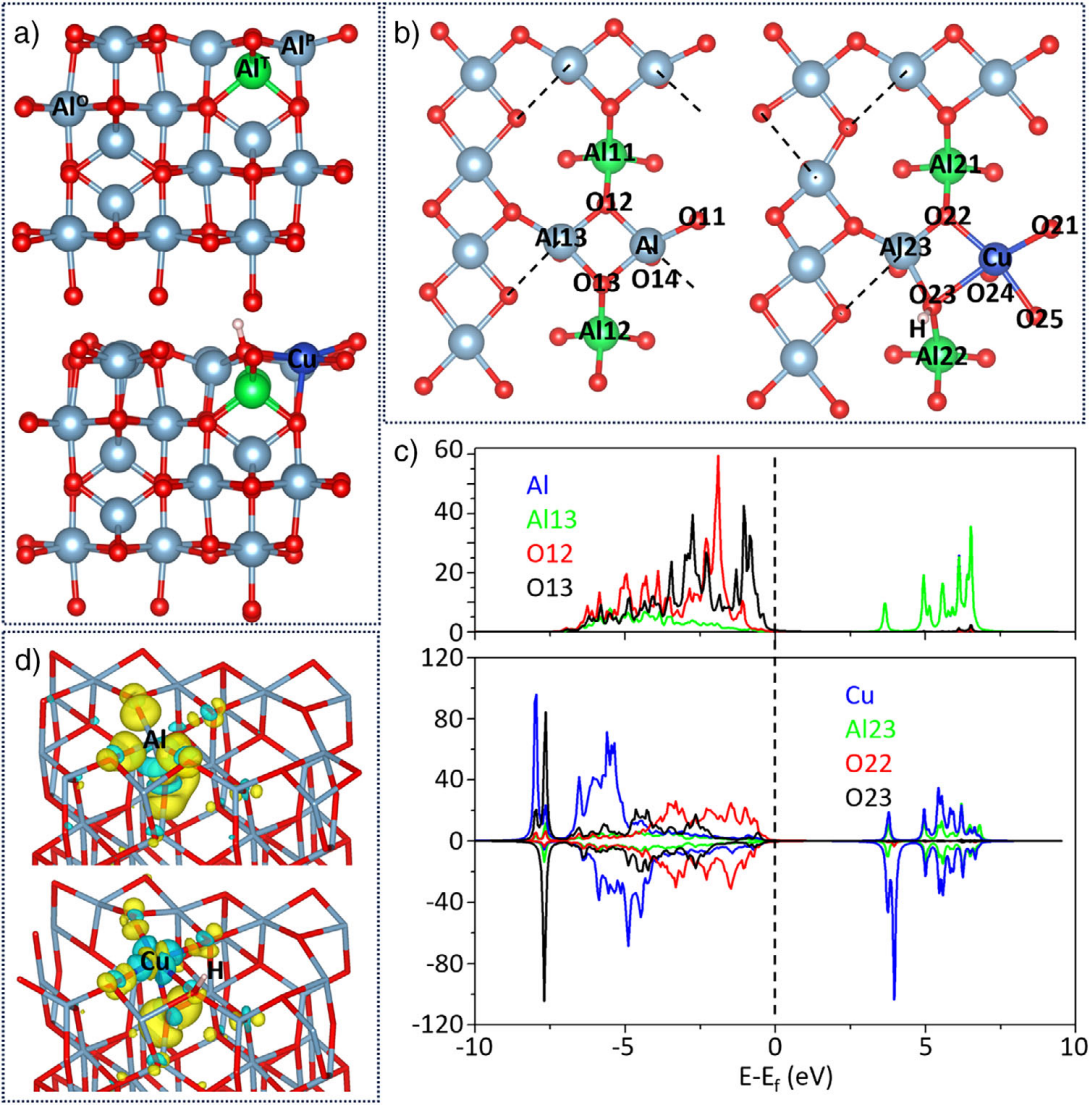
Optimized γ‐Al_2_O_3_ (100) surface and Cu‐doped (100) surface a), side view; b), top view), DOS projected onto Al and O p orbitals and Cu d orbitals c), and charge density differences as described in the text d), cyan area: losing electron; yellow area: gaining electron; isosurfaces drawn at ±0.01 a.u.).

To understand relationships between the atomic and electronic structure of these sites, we performed electronic structure calculations using density functional theory (DFT). The projected density of states (PDOS) results (Figure 4c) from our DFT calculations indicate that in the undoped γ‐Al_2_O_3_, the Al─O bonds exhibit covalent characteristics due to the strong overlap of O p orbitals with Al s and p orbitals. However, near the Fermi level (around 0 eV), the density of states for O remains relatively high, while the contribution from Al is smaller. This suggests a noticeable ionic character in Al─O bonds. Therefore, O─Al bonds in γ‐Al_2_O_3_ are better described as ionic‐covalent rather than purely ionic.^[^ ^59^, ^66^ ^]^ After Cu doping, the Cu d orbitals show significant contributions near the Fermi level, but there is minimal overlap with the density of states of O22 and O23, suggesting limited electron sharing between Cu and O. This observation supports the conclusion that the Cu─O bond has a more ionic character. The electron density difference in Figure 4d shows that in undoped γ‐Al_2_O_3_, there is a noticeable accumulation of electron density along the Al─O bond direction. This accumulation indicates electron sharing between Al and O. After Cu replaces Al, the electron density around Cu decreases (cyan regions), indicating electron transfer away from Cu. This strengthens the ionic character of the Cu─O bond while weakening the covalent nature of the Al─O bonds. This electron redistribution indicates that the unoccupied 4s and 3d orbitals of Cu on the surface could act as Lewis acid sites, exhibiting a stronger electron affinity and resulting in greater Lewis acidity compared to Al.^[^ ^67^ ^]^

To summarize the collective findings for the analyzed Cu/γ‐Al_2_O_3_ catalyst, no CuOCu^+^ peak is discernible from the ToF‐SIMS results, confirming the atomic‐level dispersion as we observed in the S/TEM characterization, CO‐IR, and XAS analysis. We conclude that the Cu/γ‐Al_2_O_3_ sample has pentahedral‐ and hydroxylated‐Cu atoms doped on the γ‐Al_2_O_3_ surface atomically (single‐atom catalyst). Additionally, the ToF‐SIMS results (Figure S3) showed that the relative contents of CuOAl^+^/Cu^+^, and CuOCu^+^/Cu^+^ in the postreaction catalyst did not change significantly, indicating that its structure remains relatively stable with no formation of Cu clusters. This confirms our initial hypothesis that a highly efficient stable 2D single‐atom Cu catalyst could be developed through a simple thermal treatment of Cu‐adsorbed γ‐AlOOH nanosheets. The core information we revealed in this work are (1) Cu single atoms substituting for Al^P^ sites on γ‐Al_2_O_3_ exhibit high CO_2_ hydrogenation activity, with 17.5% selectivity toward CH_3_OH; (2) the maximum capacity of anchoring single atoms on the alumina substrate is largely dependent on the density of surface defects; optimizing the synthesis of γ‐Al_2_O_3_ to increase Al^P^ defect density could thus be a means to enhance Cu loading and catalytic activity even further.

Given that our catalyst lacks metallic Cu^0^ sites, it is important to further investigate the mechanism of H_2_ activation. Recent work by Zheng et al. demonstrated that pentacoordinated Al^3^⁺‐enriched amorphous Al_2_O_3_ facilitates the heterolytic activation of H_2_,^[^ ^68^ ^]^ enabling the hydrogenation of substrates containing C═C and C═O bonds. This defect‐rich Al_2_O_3_ acts as a heterogeneous frustrated Lewis pair (FLP), capable of H₂ activation in the absence of any transition metal species. Therefore, it remains to be determined whether similar heterolytic H₂ dissociation occurs on our cationic Cu SAC, forming O–H^δ⁺^ and Cu–H^δ⁻^ species as the initiation of hydrogenation. In addition, activation of CO_2_ molecule is also an important prerequisite. CO_2_ molecules are known to be weakly adsorbed on the γ‐Al_2_O_3_ (110) surface with an *E*_ads_ = 0.45 eV and nearly linear configuration (∠O–C–O = 176.5°),^[^ ^69^ ^]^ and also interact with Cu^0^ weakly.^[^ ^70^, ^71^ ^]^ We observed from the charge density analysis that Cu sites are significantly electron deficient compared to Al cations; therefore, the Cu–O–Al site is hypothesized to bend the adsorbed CO_2_ molecule, thereby making it prone to receive electrons from surface H^δ⁻^. Further investigations, including ^2^H‐NMR spectroscopy, chemisorption studies, and operando infrared experiments, coupled with DFT calculations, are underway to elucidate the full hydrogenation mechanism in the reported Cu single atom catalyst.

## Conflict of Interests

The authors declare no conflict of interest.

## Supporting information



Supporting information

## Data Availability

The data that support the findings of this study are available in the Supporting Information of this article.
