# Supplementary material for: Boosting Hydrogenation of CO2 Using Cationic Cu Atomically Dispersed on 2D γ‐Al2O3 Nanosheets
Source: Angew Chem Int Ed Engl. 2025 Apr 26;64(25):e202505444. doi: 10.1002/anie.202505444 (PMC12171327; doi:10.1002/anie.202505444)
Supplement: Supplementary file 1 — Supporting information [file ANIE-64-e202505444-s001.docx]

**Boosting Hydrogenation of CO_2_ using Cationic Cu Atomically Dispersed on 2D γ-Al_2_O_3_ Nanosheets**

Ping Chen^†^, Yifeng Zhu^†^, Hailin Zhang^†^, Micah P. Prange, Duo Song, Janos Szanyi, Yining Wang, Ying Chen, Xiang Wang, Oliver Y. Gutiérrez, Zihua Zhu, Zheming Wang, Carolyn I. Pearce, Ping Li*, Kevin M. Rosso*, Honghong Shi*, Xin Zhang*

Dedication [†] These authors contributed equally to this work.

[a] Dr. P. Chen, Dr. Y. Zhu, Dr. H. Zhang, Dr. M. P. Prange, Dr. D. Song, Dr. Y. Wang, Dr. Y. Chen, Dr. X. Wang, Dr. Z. Wang, Dr. C. I. Pearce, Dr. K. M. Rosso and Dr. X. Zhang

Physical & Computational Science Directorate

Pacific Northwest National Laboratory

Richland, Washington 99354, United States

Email: [xin.zhang@pnnl.gov](mailto:xin.zhang@pnnl.gov) and kevin.rosso@pnnl.gov

Dr. H. Zhang and Prof. P. Li

Institute of Process Engineering

Chinese Academy of Sciences

Beijing 100190, People’s Republic of China

Email: lipinggnipil@ipe.ac.cn

Dr. J. Szanyi, Dr. O.Y. Gutiérrez and Dr. H. Shi

Institute of Integrated Catalysis

Pacific Northwest National Laboratory

Richland, WA 99354, United States

Email: [crystal.shi@pnnl.gov](mailto:crystal.shi@pnnl.gov)

Dr. Z. Zhu

Environmental Molecular Sciences Laboratory

Pacific Northwest National Laboratory

Richland, Washington 99354, United States

**Methods**

**Material Synthesis**

Copper nitrate (Cu(NO_3_)_2_·3H_2_O, ≥99%), sodium hydroxide (NaOH, ≥ 98%) and aluminum hydroxide (Al(OH)_3_, ≥98%) were purchased from Sigma-Aldrich Chemical Reagent Co., Ltd. All chemicals were commercially supplied as analytical reagents and used as received without further purification.

The γ-AlOOH was synthesized through a simple method using H_2_SO_4_ and NaAlO_2_ in aqueous solution.^[1]^ In a typical procedure, 26 mL of 4.5 mol/L H_2_SO_4_ was added into 400 mL of 48 g/L NaAlO_2_ solution at 25 ^o^C with vigorous stirring for 30 min. The resulting γ-AlOOH was filtered and thoroughly washed with distilled water until no SO_4_^2-^ were detected using the ultraviolet spectrophotometry.^[2]^ The material was then dried at 100 ^o^C overnight.

The catalysts were prepared by wet impregnation method. Briefly, the copper salt (Cu(NO_3_)_2_·3H_2_O) was dissolved in a small amount of distilled water and was then mixed together with γ-AlOOH. In a typical process, 100 ml 100 mg/L of Cu (II)-containing solution and 100 mg γ-AlOOH were transferred into a polytetrafluoroethylene bottles (250 ml) and then shaken at 150 rpm at room temperature for 5 h. Experiments were performed by varying initial concentrations (10-200 mg/L of Cu(II) precursor solution). Then the obtained product was filtered and dried at 100℃ overnight and then calcined at 600 ℃ for 2 h under a nitrogen atmosphere. The obtained catalysts were named Cu11, Cu22, Cu33, Cu44 (labeled as Cu/γ-Al_2_O_3_ in the main text), Cu55, respectively.

**Characterizations**

The catalysts were identified by a PANalytical Empyrean X-ray powder diffractometer (XRD) equipped with a Cu-Kα readiation source. Diffraction patterns were collected from 5-80º (using a step size of 0.026º). Surface area and pore structure were analyzed by N_2_ physisorption at 350 ºC with an automatic gas sorption system: Quadrasorb EVO/ SI Gas Sorption System from Quantachrome Instruments (A brand of Anton Paar). The samples were degassed under vacuum at 150 ºC for 12 h before the N_2_ physisorption measurements. Structural analysis was performed with aberration corrected Thermo-Fisher Spectra scanning/transmission electron microscope (S/TEM) operated at 300kV. The observations were performed in Scanning mode using a HAADF detector. The probe convergence angle was 30 mrad, and the inner detection angle on the HAADF detector of 68 mrad. The acquisition and basic image processing were performed with Thermo-Fisher’s TIA and Velox software.

^27^Al solid-state NMR spectra were acquired on a JNM-ECZ600R spectrometer by using a pulse-acquire sequence with a 90°pulse duration of 2.5 µs and a recycle interval of 5 s. ^27^Al Larmor frequency were 156.34 MHz. Single-pulse ^27^Al MAS NMR experiments were performed at 20 °C on a Varian-DDR 19.975 T (with a ^27^Al Larmor frequency of 221.413 MHz) NMR spectrometer using a commercial 3.2 mm pencil-type probe with a spinning speed of 20 kHz. The typical parameters for acquiring NMR spectra for quantitative analysis were spectrum width = 500 kHz, recycle delay time = 1 s (an array of recycle delay times from 0.5 s to 5 s confirmed that 1 s is sufficient for reaching the equilibrium state between each scan), acquisition time = 20 ms, number of scans = 5120 - 10240, and a small tip angle π/20 (corresponding to pulse with = 0.55 us). Chemical shifts were referenced to 1 M AlCl_3_ aqueous solution at 0 ppm.

Cu (II) and Al (III) ion concentrations were measured by an inductively coupled plasma optical emission spectroscopy (ICP-OES, PE Optima 7300DV, PerkinElmer). The XPS spectra of the copper-based catalyst before and after catalysis were obtained with an ESCALAab220i-XL electron spectrometer from VG Scientific using 300 W Al-Kα radiation. The aggregate form of copper was performed by ToF-SIMS instrument (IONTOF GmbH, Münster, Germany). A 25 keV pulsed Bi_3_^+^ beam was used as the analysis beam to collect SIMS spectra. The Bi_3_^+^ beam with a current of 0.56 pA and the data collection time of ∼96 s per spectrum was focused to be ∼5μm diameter and scanned over a 200×200 μm^2^ area.

The IR spectroscopy experiments were performed with a Nicolet iS50 FT-IR (ThermoScientific) spectrometer equipped with a cell and a vacuum chamber (resolution of 4 cm^-1^, 256 scans). Prior to CO adsorption, the sample pellet was in situ reduced at 250 ℃ for 30 min in 1 torr of H_2_, cool down to room temperature and evacuated. Then, the pellet was exposed to CO up to 3.5 Torr at -173 ℃, then the cell was heated up slowly in CO back to close to room temperature (~9 ℃) to collect the spectra in Supplementary Fig. S4 and evacuated at room temperature to collect the spectra in Fig. 3h.

X-ray adsorption spectra at the Cu K-edge was measured at Sector 20 of the Advanced Photon Source operated by Argonne National Laboratory. The samples were H_2_-pretreated (as described for the reaction) then transferred to sealed capillaries under air-free conditions prior to the measurements. The edge positions were calibrated based on the spectra of Cu foils. The ATHENA and ARTEMIS programs were used to analyze data from the EXAFS and X-ray absorption near edge spectroscopy measurements. Cu k-edge EXAFS data were fitted to the theoretical structural models derived using the FEFF9 code with the following details. The quantitative curve-fitting was performed using the ARTEMIS module of the IFEFFIT software package. The amplitude reduction factor S0^2^ and the edge-energy shift ∆E_0_ were determined to be 0.811 and 4.039 eV respectively (obtained from the fitting of Cu(OH)_2_ standard material). Fourier Transform parameters ranges: 1.0 ≤ *k* ≤ 8.5 Å^-1^, 1.0 ≤ *R* ≤ 3.4 Å. The CN and internal atomic distance changes (∆R) were allowed to vary as separate parameters for each path. The *R* factor is 2.9% for this fit demonstrating a satisfying curve fitting quality. The Artemis input files containing the information on γ-Al_2_O_3_ crystal structure, lattice parameters and space group were taken from literature.^[3]^ The distances for Cu-O and Cu-OH are from the FEFF file of Cu(OH)_2_ crystal structure. The distances for Cu-O-Al^T^ and Cu-Al^O^ are from the FEFF file of γ-Al_2_O_3_ crystal structure of selecting the Al^P^ as the core-atom and its substitution into the Cu atom.

**Catalytic tests**

Prior to the reaction, these catalysts were reduced in 20 vol% H_2_ carried by N_2_ at 250 ℃ for 30 min with a total flow rate of 30 ml/min. The performance of Cu/γ-Al_2_O_3_ catalysts in CO_2_ hydrogenation was evaluated at 250 ℃ and 32 bar in a stainless steel fixed-bed reactor. GHSV=72500 mL/h/g. The mixture gas (V_H2_/V_CO2_/V_N2_ = 21/7/1 mL/min) was fed through the reactor with a 29 mL/min flow rate. The gas samples were analyzed by an online gas chromatograph equipped with an FID for methanol and TCD for N_2_, CO, and CH_4_. For the temperature dependence studies, the activity and selectivity of CO₂, CO, and methanol were determined using internal standards, based on the average of three steady-state measurements, with duration at each temperature lasting ~150 minutes. No significant induction period or deactivation was observed during these tests. After finishing the tests at six individual temperatures (Fig S1), the used Cu/γ-Al_2_O_3_ catalyst (Cu44) are transferred to the glovebox and subsequently characterized using ToF-SIMS, with the results presented in Figure S3. The cumulative on-stream time of the used Cu/γ-Al_2_O_3_ catalyst is approximately ~1000 minutes.

**DFT calculations**

All DFT calculations were conducted using the pseudopotential plane-wave DFT approach implemented in the NWPW module of the NWChem computational chemistry package.^[4]^ The Perdue-Burke-Ernzerhof exchange-correlation functional (PBE96) was utilized throughout the PSPW optimization procedure.^[5]^ The DFT + U approach was applied to account for the electron correlation in the localized Cu 3d orbitals.^[6]^ And the effective on-site Coulomb interaction parameter for each Cu atom was set to 7 eV.^[7]^ The Grimme DFT-D3 method for long-range dispersion interactions was also used in the calculations.^[8]^ Default pseudopotentials contained in NWChem were used. All the pseudopotentials were modified to the separable form suggested by Kleinman and Bylander.^[9]^ Periodic boundary conditions were used and wavefunction solutions were obtained at the Γ-point with a wavefunction cutoff energy of 100 Ry and a density cutoff energy of 200 Ry. All atomic positions were entirely relaxed in a fixed unit cell using the default NWChem DRIVER optimizer until the forces on the atoms were converged to 10^-2^ eV/Å and the total energy was converged to 10^-5^ eV.

The (100) surface model for γ-Al_2_O_3_ was taken from the work of Digne et al.^[10]^ Although their non-spinel model remains a subject of significant debate,^[3, 11]^ it has been applied in numerous studies,^[12]^ particularly those focusing on Cu substitution.^[6, 13]^ In this work, only the (100) surface was chosen because the (100) and (110) surfaces make up 90% of the γ-Al_2_O_3_ surface,^[10a, 14]^ but the (110) surface exposes a noticeable amount of tri-coordinated Al,^[15]^ which does not align with the ^27^Al MAS NMR results in this study. And the (100) surface contains both Al^T^ and Al^P^ sites, which perfectly matches our ^27^Al MAS NMR observations (Fig. 1b, Table S3). Most importantly, the Cu-doped (100) surface model shows excellent agreement with our EXAFS fitting results (Fig. 3j, Table S5). Therefore, the simulations were carried out by using the unit cell (a = 5.587 Å, b = 8.413 Å, c = 8.068 Å, β = 90.59°). The (100) surface slab was created by expanding the conventional unit cell doubly along a and c directions, resulting in a (2×1) surface slab (a = 11.174 Å, b = 8.413 Å) with 13 Al layers. All the slabs consist of an upper and a lower surface with the same outmost atomic layer and a vacuum of approximately 10 Å in between. The (100) surface slab consisted of 64 Al and 96 O with the thickness of slab 16.136 Å, which corresponds to the distance in c direction between two oxygen atoms on surface. For Cu/γ-Al_2_O_3_, a reaction formula is proposed for the substitution of a surface Al atom by Cu and H atoms, considering charge balance.^[6]^ The optimal locations for the Cu and H atoms on the surface were determined based on EXAFS fitting results. The two surfaces were modelled by fully relaxed slabs without inversion symmetry. Finally, Mulliken population analyses were performed using the same level of theory to calculate the atom-projected density of states. For the charge density difference (Δρ) calculation, we use the studied atom and its underlying bonding oxygen atoms as the central unit. The formula is expressed as:

Δ𝜌=𝜌_slab_ − (𝜌_slab without X-O_ + 𝜌_X-O_)

Where 𝜌_slab_ represents the total charge density of the slab, 𝜌_slab without X-O_ represents the charge density of the slab with the central unit (X-O) removed, and ρ_X-O_ represents the charge density of only the central unit (X-O). The calculated charge density difference can be visualized using graphical tools-VESTA.

**Table S1**. Physisorption analysis results of γ-AlOOH

|  | Surface area (m^2^/g)^a^ | Total pore volume (cc/g)^b^ | Average pore diameter (nm)^c^ |
| --- | --- | --- | --- |
| γ-AlOOH | 349 | 0.70 | 5.6 |
| γ-Al_2_O_3_ | 185 | 0.79 | 9.4 |
| Cu/γ-AlOOH | 332 | 0.84 | 5.5 |
| Cu/γ-Al_2_O_3_ | 282 | 0.83 | 6.5 |

^a^ Surface area was calculated by the Brunner-Emmet-Teller method.

^b^ Total pore volume was obtained over the P/P0 value of 0.996.

^c^ Average pore diameter was calculated by the Barrett-Joyner-Halenda method.

Table S2. CO_2_ hydrogenation results on Cu-doping γ-Al_2_O_3_ catalysts.

|  | Cu loading (wt.%) | CO_2_ rate (mol/mol/h) | CO rate (mol/mol/h) | MeOH+DME (mol/mol/h) |
| --- | --- | --- | --- | --- |
| Cu11 | 0.244 | 16.65 | 16.36 | 0.29 |
| Cu22 | 0.362 | 27.77 | 23.74 | 4.03 |
| Cu33 | 0.432 | 28.05 | 23.51 | 4.54 |
| Cu44 | 0.486 | 30.45 | 25.10 | 5.35 |
| Cu55 | 0.525 | 24.01 | 18.87 | 5.15 |

Reaction conditions: 250 ^o^C, 32 bar, CO_2_/H_2_/N_2_ = 7/21/1 mL/min, 24 mg catalyst, 200 mg SiC; the Cu loadings (wt%) are measured by Inductively coupled plasma optical emission spectroscopy (ICP-OES).


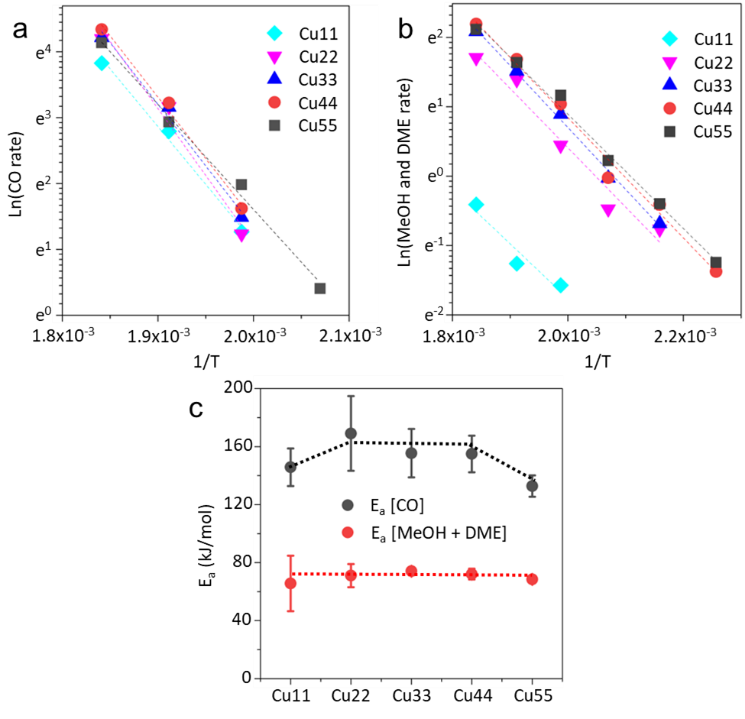


**Fig. S1**. Effects of temperature on (a) rates of CO (based on TOF in h^-1^), (b) rates of MeOH + DME (based on TOF in h^-1^), (c) calculated activation energy (Ea, kJ/mol).


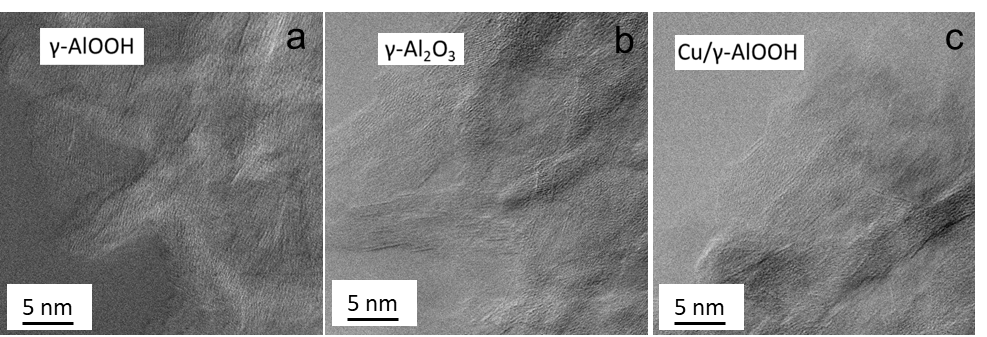


**Fig. S2.** TEM images of γ-AlOOH, γ-Al_2_O_3_, and Cu/γ-AlOOH.

Table S3. The percentage of Al^O^, Al^P^ and Al^T^ in γ-AlOOH, γ-Al_2_O_3_, Cu/γ-AlOOH, and Cu/γ-Al_2_O_3_

| mol% | Al^O^ (10.8 ppm) | Al^P^ (36.6 ppm) | Al^T^ (70.1 ppm) |
| --- | --- | --- | --- |
| γ-AlOOH | 99.1 | 0.5 | 0.4 |
| γ-Al_2_O_3_ | 76.4 | 0.7 | 22.9 |
| Cu/γ-AlOOH | 99.4 | 0.3 | 0.3 |
| Cu/γ-Al_2_O_3_ | 70.5 | 0.1 | 29.4 |

Table S4. O1s peak fitting results from XPS spectra

|  | O^2-^ (eV) | FWHM | Area (%) | OH (eV) | | FWHM | | Area (%) | H_2_O (eV) | FWHM | Area (%) |
| --- | --- | --- | --- | --- | --- | --- | --- | --- | --- | --- | --- |
| γ-AlOOH | 530.7 | 1.8 | 37.4 | 532.1 | 1.8 | | 55.0 | | 533.5 | 1.8 | 7.5 |
| Cu/γ-AlOOH | 530.7 | 1.5 | 25.1 | 532.0 | 1.8 | | 50.9 | | 533.1 | 1.8 | 24.0 |
| γ-Al_2_O_3_ | 530.8 | 1.6 | 33.8 | 532.2 | 1.7 | | 59.2 | | 533.4 | 1.8 | 7.0 |
| Cu/γ-Al_2_O_3_ | 530.8 | 1.7 | 29.7 | 532.0 | 1.6 | | 43.1 | | 533.3 | 1.8 | 27.2 |


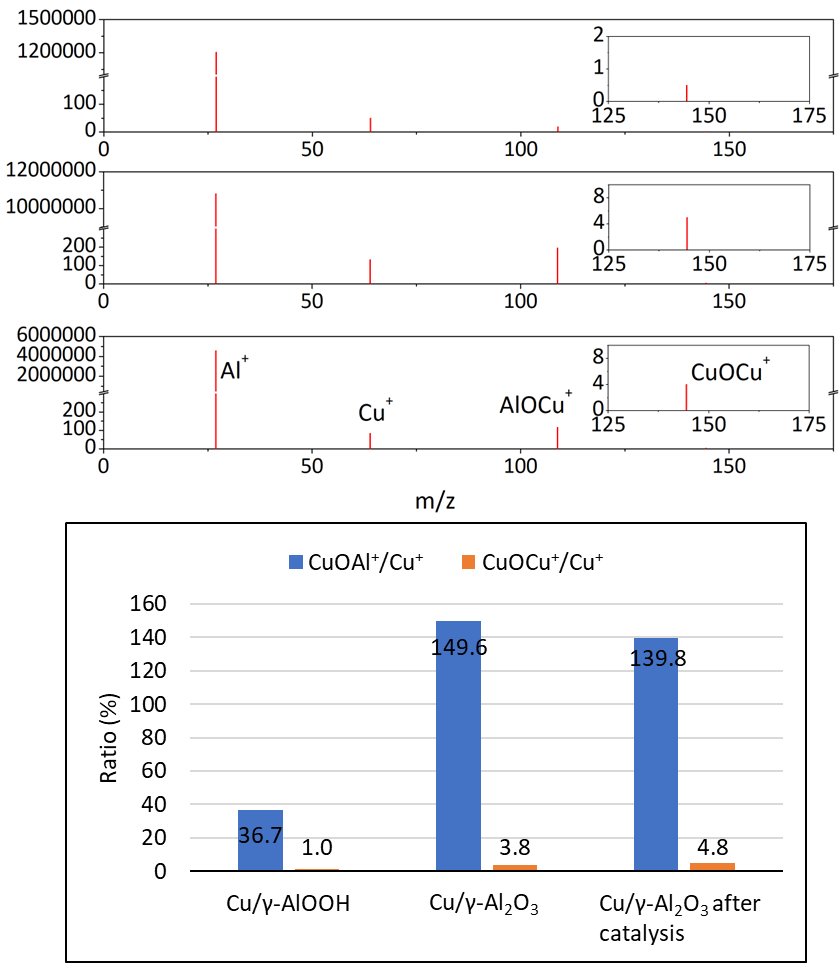


**Fig. S3.** Positive ion ToF-SIMS spectra of γ-AlOOH and Cu/γ-Al_2_O_3_ before and after catalysis, and their normalized relative ratios.


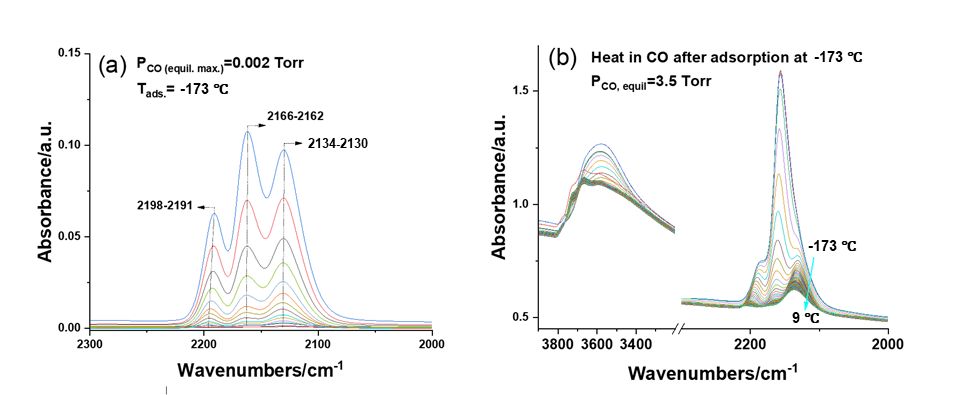


**Fig. S4.** FTIR spectra of CO adsorption on the studied Cu/γ-Al_2_O_3_ catalyst. (a) CO adsorption at -173 ℃. (b) heating after CO adsorption.


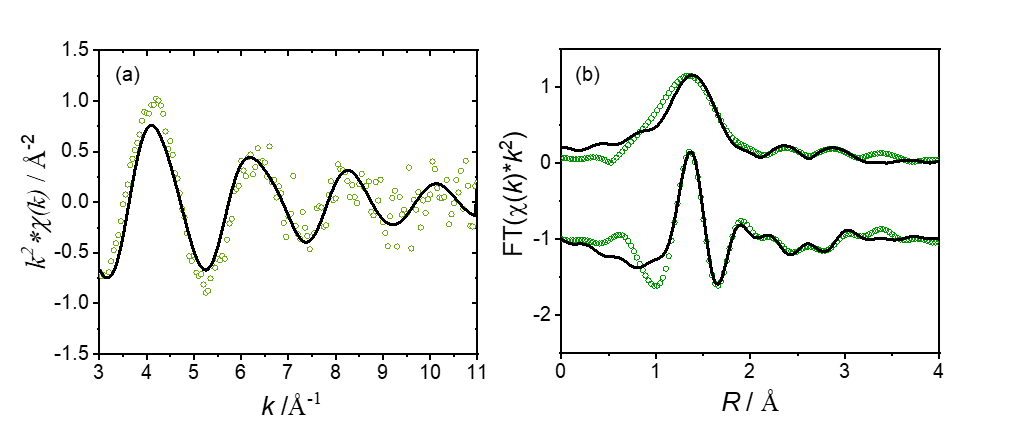


**Fig. S5**. Cu K-edge EXAFS (points) and the curve fit (line) for the Cu/γ-Al_2_O_3_: (a) shown in k^2^-weighted *k*-space; (b) shown in *R* space. The data are k^2^-weighted and not phase corrected.

**
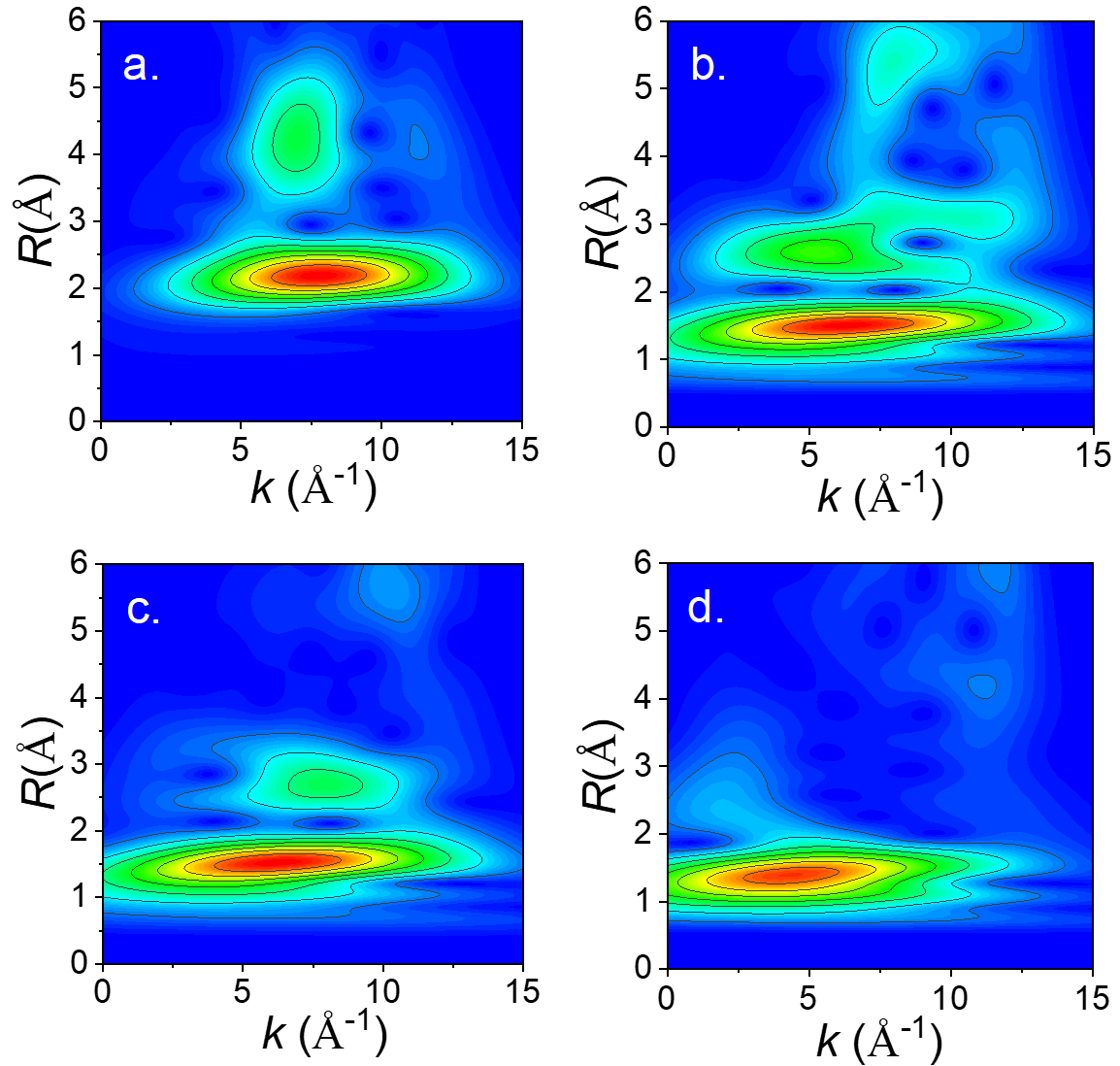
**

**Fig. S6.** Wavelet-transform analysis of the Cu K-edge EXAFS spectra. (a) Cu foil reference (b) CuO reference (c) Cu(OH)_2_ reference (d) Cu/γ-Al_2_O_3_ catalyst. The Morlet function is used in this wavelet-transform analysis with two parameters as κ = 10 and σ = 1.

Table S5. Summary of EXAFS fitting results

| Sample | Path | ΔR / Å | R / Å | N | σ^2^/ Å^2^ |
| --- | --- | --- | --- | --- | --- |
| Cu/γ-Al_2_O_3_ | Cu-O | -0.03 ± 0.01 | 1.97 | 3.3 ± 0.8 | 0.004 ± 0.003 |
|  | Cu-OH | -0.03 ± 0.01 | 2.27 | 0.8 ± 0.2 | 0.004 ± 0.003 |
|  | Cu-O-Al^O/P^ | -0.05 ± 0.04 | 2.81 | 3.5 ± 1.7 | 0.020 |
|  | Cu-O-O | -0.06 ± 0.02 | 3.13 | 10.0 ± 2.3 | 0.008 ± 0.006 |
|  | Cu-O-Al^T^ | 0.10 ± 0.06 | 3.25 | 3.9 ± 2.2 | 0.020 |

Table S6. Optimized bond length parameters (unit: Å) based on Fig. 4.

| γ-Al_2_O_3_ | Al-Al^T^11 | Al-Al^T^12 | Al-Al^P^13 | Al-O11 | Al-O12 | Al-O13 | Al-O14 |  |
| --- | --- | --- | --- | --- | --- | --- | --- | --- |
|  | 3.15 | 3.22 | 2.72 | 1.85 | 1.84 | 1.91 | 1.79 |  |
| Cu/γ-Al_2_O_3_ | Cu-Al^T^21 | Cu-Al^T^22 | Cu-Al^P^23 | Cu-O21 | Cu-O22 | Cu-O23(H) | Cu-O24 | Cu-O25 |
|  | 3.30 | 3.53 | 2.85 | 2.02 | 1.99 | 2.40 | 2.03 | 2.11 |
| Note: A Cu-Al^O^ distance at 2.86 Å was not displaced in Fig. 4, refer to Cu-O25-Al^O^.  See cif files Appendix 1 and 2 | | | | | | | | |

**Appendix 1. CIF file of γ-Al_2_O_3_ (110) surface**

data_nwchem_pspw

_audit_creation_date Fri Sep 6 03:58:17 2024

_audit_creation_method generated by PSPW module of NWChem

_cell_length_a 11.1740

_cell_length_b 8.4130

_cell_length_c 27.0000

_cell_angle_alpha 90.0000

_cell_angle_beta 90.5900

_cell_angle_gamma 90.0000

_symmetry_space_group_name_H-M P1

loop_

_atom_site_type_symbol

_atom_site_fract_x

_atom_site_fract_y

_atom_site_fract_z

Al 0.239023 -0.250000 0.067226

Al -0.138763 0.250000 -0.007363

Al -0.010051 0.250000 0.176437

Al 0.109806 -0.250000 -0.116625

Al -0.013199 -0.250000 0.066730

Al 0.113351 0.250000 -0.006904

Al -0.143536 -0.250000 -0.046214

Al 0.243612 0.250000 0.106055

Al 0.233845 0.075741 -0.085739

Al -0.133639 -0.075755 0.145561

Al -0.133639 -0.424246 0.145560

Al 0.233845 0.424259 -0.085739

Al 0.108040 -0.421941 -0.010995

Al -0.007823 0.421970 0.070901

Al -0.007823 0.078031 0.070901

Al 0.108040 -0.078059 -0.010995

O -0.010097 -0.250000 -0.007727

O 0.110190 0.250000 0.067646

O -0.141538 0.250000 -0.076807

O 0.242071 -0.250000 0.136640

O 0.231187 -0.250000 -0.003044

O -0.130810 0.250000 0.063016

O 0.118652 0.250000 -0.081126

O -0.018225 -0.250000 0.140803

O -0.005499 0.405419 -0.000229

O 0.105762 -0.405435 0.060129

O 0.105762 -0.094565 0.060129

O -0.005499 0.094581 -0.000229

O -0.148808 -0.082347 -0.084972

O 0.248671 0.082075 0.144689

O 0.248671 0.417926 0.144688

O -0.148808 -0.417654 -0.084972

O 0.228913 0.406058 -0.013360

O -0.128786 -0.406125 0.073127

O -0.128786 -0.093875 0.073127

O 0.228913 0.093942 -0.013360

O 0.116491 -0.081430 -0.078031

O -0.016242 0.081283 0.137952

O -0.016242 0.418718 0.137952

O 0.116491 -0.418571 -0.078031

Al 0.222989 -0.250000 -0.225453

Al -0.123323 0.250000 0.285130

Al -0.013825 0.250000 -0.118093

Al 0.114031 -0.250000 0.177847

Al -0.021541 -0.250000 -0.226872

Al 0.121157 0.250000 0.286442

Al -0.164792 -0.250001 0.255991

Al 0.264853 0.250001 -0.196215

Al 0.236118 0.073066 0.212702

Al -0.136294 -0.073186 -0.152986

Al -0.136293 -0.426814 -0.152986

Al 0.236118 0.426934 0.212702

Al 0.082201 -0.411789 0.281549

Al 0.017684 0.411758 -0.221847

Al 0.017685 0.088245 -0.221847

Al 0.082199 -0.088214 0.281549

O -0.026371 -0.250002 0.289855

O 0.126461 0.250002 -0.230134

O -0.134976 0.250001 0.217950

O 0.234626 -0.250000 -0.158214

O 0.198552 -0.250000 0.294010

O -0.098576 0.250000 -0.234382

O 0.122835 0.250000 0.215082

O -0.023095 -0.250000 -0.155463

O 0.006304 0.400780 0.298457

O 0.093229 -0.400785 -0.239006

O 0.093228 -0.099214 -0.239006

O 0.006305 0.099218 0.298457

O -0.151541 -0.086169 0.213733

O 0.251766 0.086289 -0.153885

O 0.251767 0.413712 -0.153885

O -0.151541 -0.413832 0.213733

O 0.241337 0.400519 0.285586

O -0.141617 -0.400533 -0.225922

O -0.141620 -0.099471 -0.225922

O 0.241340 0.099485 0.285587

O 0.112608 -0.078284 0.216609

O -0.012883 0.078233 -0.156870

O -0.012882 0.421768 -0.156870

O 0.112607 -0.421717 0.216609

Al -0.260977 -0.250000 0.067226

Al 0.361237 0.250000 -0.007363

Al 0.489949 0.250000 0.176437

Al -0.390194 -0.250000 -0.116625

Al 0.486801 -0.250000 0.066730

Al -0.386649 0.250000 -0.006904

Al 0.356464 -0.250000 -0.046214

Al -0.256388 0.250000 0.106055

Al -0.266155 0.075741 -0.085739

Al 0.366361 -0.075755 0.145561

Al 0.366361 -0.424246 0.145560

Al -0.266155 0.424259 -0.085739

Al -0.391960 -0.421941 -0.010995

Al 0.492177 0.421970 0.070901

Al 0.492177 0.078031 0.070901

Al -0.391960 -0.078059 -0.010995

O 0.489903 -0.250000 -0.007727

O -0.389810 0.250000 0.067646

O 0.358462 0.250000 -0.076807

O -0.257929 -0.250000 0.136640

O -0.268813 -0.250000 -0.003044

O 0.369190 0.250000 0.063016

O -0.381348 0.250000 -0.081126

O 0.481775 -0.250000 0.140803

O 0.494501 0.405419 -0.000229

O -0.394238 -0.405435 0.060129

O -0.394238 -0.094565 0.060129

O 0.494501 0.094581 -0.000229

O 0.351192 -0.082347 -0.084972

O -0.251329 0.082075 0.144689

O -0.251329 0.417926 0.144688

O 0.351192 -0.417654 -0.084972

O -0.271087 0.406058 -0.013360

O 0.371214 -0.406125 0.073127

O 0.371214 -0.093875 0.073127

O -0.271087 0.093942 -0.013360

O -0.383509 -0.081430 -0.078031

O 0.483758 0.081283 0.137952

O 0.483758 0.418718 0.137952

O -0.383509 -0.418571 -0.078031

Al -0.277011 -0.250000 -0.225453

Al 0.376677 0.250000 0.285130

Al 0.486175 0.250000 -0.118093

Al -0.385969 -0.250000 0.177847

Al 0.478459 -0.250000 -0.226872

Al -0.378843 0.250000 0.286442

Al 0.335208 -0.250001 0.255991

Al -0.235147 0.250001 -0.196215

Al -0.263882 0.073066 0.212702

Al 0.363706 -0.073186 -0.152986

Al 0.363707 -0.426814 -0.152986

Al -0.263882 0.426934 0.212702

Al -0.417799 -0.411789 0.281549

Al -0.482316 0.411758 -0.221847

Al -0.482315 0.088245 -0.221847

Al -0.417801 -0.088214 0.281549

O 0.473629 -0.250002 0.289855

O -0.373539 0.250002 -0.230134

O 0.365024 0.250001 0.217950

O -0.265374 -0.250000 -0.158214

O -0.301448 -0.250000 0.294010

O 0.401424 0.250000 -0.234382

O -0.377165 0.250000 0.215082

O 0.476905 -0.250000 -0.155463

O -0.493696 0.400780 0.298457

O -0.406771 -0.400785 -0.239006

O -0.406772 -0.099214 -0.239006

O -0.493695 0.099218 0.298457

O 0.348459 -0.086169 0.213733

O -0.248234 0.086289 -0.153885

O -0.248233 0.413712 -0.153885

O 0.348459 -0.413832 0.213733

O -0.258663 0.400519 0.285586

O 0.358383 -0.400533 -0.225922

O 0.358380 -0.099471 -0.225922

O -0.258660 0.099485 0.285587

O -0.387392 -0.078284 0.216609

O 0.487117 0.078233 -0.156870

O 0.487118 0.421768 -0.156870

O -0.387393 -0.421717 0.216609

**Appendix 2. CIF file of Cu-Doped γ-Al_2_O_3_ (110) surface**

data_nwchem_pspw

_audit_creation_date Fri Sep 20 18:29:21 2024

_audit_creation_method generated by PSPW module of NWChem

_cell_length_a 11.1740

_cell_length_b 8.4130

_cell_length_c 27.0000

_cell_angle_alpha 90.0000

_cell_angle_beta 90.5900

_cell_angle_gamma 90.0000

_symmetry_space_group_name_H-M P1

loop_

_atom_site_type_symbol

_atom_site_fract_x

_atom_site_fract_y

_atom_site_fract_z

Al -0.460694 -0.254097 0.067046

Al 0.161044 0.246067 -0.007770

Al 0.289287 0.246722 0.176204

Al 0.409459 -0.253951 -0.116920

Al 0.287061 -0.253489 0.066237

Al 0.413569 0.246174 -0.007383

Al 0.156133 -0.253931 -0.046495

Al -0.456374 0.246549 0.106000

Al -0.466224 0.071728 -0.086066

Al 0.166757 -0.077012 0.144458

Al 0.166655 -0.428396 0.143861

Al -0.466364 0.420171 -0.086005

Al 0.408074 -0.425632 -0.011355

Al 0.292726 0.417899 0.070175

Al 0.292800 0.074853 0.070296

Al 0.408196 -0.081887 -0.011322

O 0.289679 -0.253786 -0.008075

O 0.410980 0.246395 0.067057

O 0.158056 0.245435 -0.077120

O -0.457228 -0.255058 0.136485

O -0.468961 -0.254039 -0.003345

O 0.169300 0.246538 0.062416

O 0.418506 0.245719 -0.081449

O 0.282083 -0.254565 0.139748

O 0.294881 0.401610 -0.000820

O 0.405932 -0.409101 0.059715

O 0.406105 -0.098200 0.059782

O 0.294809 0.091029 -0.000713

O 0.150494 -0.086479 -0.085322

O -0.451227 0.080085 0.145085

O -0.450661 0.414655 0.144706

O 0.150894 -0.421854 -0.085103

O -0.470956 0.402217 -0.013759

O 0.171399 -0.410085 0.072096

O 0.171671 -0.096649 0.072401

O -0.470984 0.090076 -0.013695

O 0.416100 -0.085225 -0.078373

O 0.284154 0.079127 0.137159

O 0.284274 0.414761 0.137144

O 0.416312 -0.422328 -0.078332

Al -0.477653 -0.255003 -0.225708

Al 0.176464 0.247102 0.285203

Al 0.285858 0.245363 -0.118343

Al 0.414055 -0.256302 0.177128

Al 0.277911 -0.254160 -0.227034

Al 0.417962 0.244413 0.287050

Al 0.148809 -0.252325 0.250947

Al -0.435927 0.245894 -0.196534

Al -0.467056 0.066675 0.213284

Al 0.162591 -0.077832 -0.153373

Al 0.163678 -0.431628 -0.153013

Al -0.464337 0.421166 0.212362

Al 0.382955 -0.410034 0.281525

Al 0.315538 0.408888 -0.222183

Al 0.318176 0.085202 -0.221964

Al 0.391289 -0.081810 0.281659

O 0.276897 -0.246682 0.289759

O 0.425539 0.247151 -0.230424

O 0.163581 0.245940 0.217651

O -0.465614 -0.255351 -0.158505

O -0.498910 -0.258376 0.293965

O 0.200665 0.245009 -0.234571

O 0.422000 0.248280 0.215142

O 0.276471 -0.254189 -0.155680

O 0.310234 0.403578 0.298426

O 0.393407 -0.404374 -0.239286

O 0.391862 -0.102854 -0.239105

O 0.295631 0.101352 0.299146

O 0.152366 -0.085673 0.212517

O -0.449400 0.081556 -0.154394

O -0.448322 0.408979 -0.154040

O 0.153885 -0.419211 0.212139

O -0.454780 0.385716 0.288146

O 0.159782 -0.407585 -0.225922

O 0.156026 -0.106437 -0.226375

O -0.483246 0.072638 0.284386

O 0.409947 -0.086179 0.214906

O 0.286219 0.073487 -0.157205

O 0.287130 0.417366 -0.156966

O 0.414273 -0.427064 0.216716

Al 0.039228 -0.253182 0.066482

Al -0.338714 0.246221 -0.007536

Al -0.211157 0.246161 0.177240

Al -0.090769 -0.253860 -0.116848

Al -0.213454 -0.253492 0.066641

Al -0.086854 0.246398 -0.007001

Al -0.343800 -0.254054 -0.046509

Al 0.043746 0.247147 0.105695

Al 0.033223 0.071769 -0.086013

Al -0.333824 -0.078431 0.145784

Al -0.333008 -0.427277 0.145851

Al 0.033063 0.420385 -0.085925

Al -0.092748 -0.425728 -0.011174

Al -0.207908 0.418434 0.071072

Al -0.207818 0.074819 0.071027

Al -0.092816 -0.081479 -0.011249

O -0.210779 -0.253661 -0.007822

O -0.089843 0.246690 0.067469

O -0.341696 0.245427 -0.077094

O 0.044063 -0.253497 0.135545

O 0.030434 -0.253659 -0.003484

O -0.330597 0.246354 0.063029

O -0.081790 0.245845 -0.081356

O -0.218297 -0.253896 0.140573

O -0.205624 0.401771 -0.000351

O -0.094421 -0.408752 0.059965

O -0.094572 -0.097970 0.059974

O -0.205674 0.091220 -0.000318

O -0.349156 -0.086657 -0.085387

O 0.048591 0.080139 0.144311

O 0.048839 0.414905 0.144388

O -0.348770 -0.421870 -0.085179

O 0.028542 0.402357 -0.013683

O -0.328529 -0.410068 0.073021

O -0.328850 -0.096967 0.073207

O 0.028457 0.090189 -0.013614

O -0.084573 -0.085002 -0.078312

O -0.216176 0.078484 0.138255

O -0.216052 0.415158 0.138455

O -0.084296 -0.422191 -0.078204

Al 0.022322 -0.254991 -0.225641

Al -0.313104 0.280640 0.286259

Al -0.214120 0.245401 -0.118393

Al -0.084007 -0.249187 0.176302

Al -0.222044 -0.254155 -0.227134

Al -0.078110 0.259887 0.288288

Al -0.363380 -0.249577 0.256415

Al 0.063964 0.245984 -0.196463

Al 0.032964 0.067731 0.212695

Al -0.337237 -0.077916 -0.153456

Al -0.336168 -0.431590 -0.153105

Al 0.039491 0.422327 0.212739

Al -0.126853 -0.396839 0.277151

Al -0.184418 0.408909 -0.222259

Al -0.181719 0.085248 -0.222027

Cu -0.116226 -0.060027 0.288297

O -0.227003 -0.245122 0.291123

O -0.074383 0.247207 -0.230481

O -0.335937 0.246821 0.219794

O 0.034243 -0.255287 -0.158416

O 0.019130 -0.279486 0.296827

O -0.299269 0.244984 -0.234658

O -0.078583 0.250665 0.216208

O -0.223472 -0.254190 -0.155780

O -0.188000 0.423998 0.296525

O -0.106506 -0.404353 -0.239327

O -0.108068 -0.102835 -0.239144

O -0.204804 0.138967 0.307824

O -0.352208 -0.088996 0.213425

O 0.050361 0.081602 -0.154344

O 0.051409 0.409069 -0.153966

O -0.349819 -0.418748 0.214835

O 0.050219 0.392962 0.286680

O -0.340143 -0.407620 -0.226029

O -0.343941 -0.106465 -0.226465

O 0.032438 0.094777 0.285943

O -0.079772 -0.086745 0.215388

O -0.213697 0.073538 -0.157279

O -0.212757 0.417367 -0.157044

O -0.079585 -0.418475 0.216025

H 0.053430 -0.305670 0.328147

**REFERENCES**

[1] P. Li, S. Zheng, P. Qing, Y. Chen, L. Tian, X. Zheng, Y. Zhang, *Green Chem.* **2014**, *16*, 4214-4222.

[2] K. Cirik, N. Dursun, E. Sahinkaya, Ö. Çinar, *Bioresour. Technol.* **2013**, *133*, 414-420.

[3] L. Kovarik, M. Bowden, K. Khivantsev, J. H. Kwak, J. Szanyi, *Acta Mater.* **2024**, *266*, 119639.

[4] a) M. Valiev, E. J. Bylaska, N. Govind, K. Kowalski, T. P. Straatsma, H. J. J. Van Dam, D. Wang, J. Nieplocha, E. Aprà, T. L. Windus, *Comput. Phys. Commun.* **2010**, *181*, 1477-1489; b) E. Apra, E. J. Bylaska, W. A. De Jong, N. Govind, K. Kowalski, T. P. Straatsma, M. Valiev, H. J. van Dam, Y. Alexeev, J. Anchell, *J. Chem. Phys.* **2020**, *152*.

[5] J. P. Perdew, K. Burke, M. Ernzerhof, *Phys. Rev. Lett.* **1996**, *77*, 3865.

[6] L. Shi, Y. Huang, Z.-H. Lu, W. Cen, X. Yu, S. Qing, Z. Gao, R. Zhang, G. Feng, *Appl. Surf. Sci.* **2021**, *535*, 147651.

[7] C. H. Kim, S. Baidya, H. Cho, V. V. Gapontsev, S. V. Streltsov, D. I. Khomskii, J.-G. Park, A. Go, H. Jin, *Phys. Rev. B* **2019**, *100*, 161104.

[8] S. Grimme, S. Ehrlich, L. Goerigk, *J. Comput. Chem.* **2011**, *32*, 1456-1465.

[9] L. Kleinman, D. Bylander, *Phys. Rev. Lett.* **1982**, *48*, 1425.

[10] a) M. Digne, P. Sautet, P. Raybaud, P. Euzen, H. Toulhoat, *J. Catal.* **2004**, *226*, 54-68; b) M. Digne, P. Sautet, P. Raybaud, P. Euzen, H. Toulhoat, *J. Catal.* **2002**, *211*, 1-5.

[11] R. Prins, *J. Catal.* **2020**, *392*, 336-346.

[12] a) J. H. Kwak, J. Hu, D. Mei, C.-W. Yi, D. H. Kim, C. H. Peden, L. F. Allard, J. Szanyi, *Science* **2009**, *325*, 1670-1673; b) R. Zhang, H. Liu, B. Wang, L. Ling, *Appl. Catal. B* **2012**, *126*, 108-120.

[13] a) L. Fu, H. Yang, *J. Phys. Chem. C* **2014**, *118*, 14299-14315; b) R. Zhang, B. Wang, H. Liu, L. Ling, *J. Phys. Chem. C* **2011**, *115*, 19811-19818.

[14] X. Krokidis, P. Raybaud, A.-E. Gobichon, B. Rebours, P. Euzen, H. Toulhoat, *J. Phys. Chem. B* **2001**, *105*, 5121-5130.

[15] Y.-X. Jin, X.-Y. Zhang, R.-X. Zhang, W.-J. Wang, J.-X. Zhao, L. Liu, W. Huang, Z.-J. Zuo, *Appl. Surf. Sci.* **2024**, *649*, 159129.
